# Role of Adiponectin and Tumor Necrosis Factor-Alpha in the Pathogenesis and Evolution of Type 1 Diabetes Mellitus in Children and Adolescents

**DOI:** 10.3390/diagnostics10110945

**Published:** 2020-11-13

**Authors:** Csilla Enikő Szabo, Oana Iulia Man, Alexandru Istrate, Eva Kiss, Andreea Catana, Victoria Creț, Radu Sorin Șerban, Ioan Victor Pop

**Affiliations:** 1 Department of Pediatrics Clinic I, University of Medicine and Pharmacy “Iuliu Hațieganu”, Victor Babeș street 8, 400012 Cluj-Napoca, Romania; ev_kiss@yahoo.co.uk (E.K.); radusorinserb@yahoo.com (R.S.Ș.); 2 Department of Pediatrics Clinic I, Emergency Clinic Hospital for Children, Motilor street 68, 400370 Cluj-Napoca, Romania; oannaaman@yahoo.com (O.I.M.); victoria_cret@yahoo.com (V.C.); 3 Department of Epidemiology, Clinical Hospital of Infectious Diseases, Iuliu Moldovan street 23, 400348 Cluj-Napoca, Romania; alex.istrate.ro@gmail.com; 4 Department of Medical Genetics, University of Medicine and Pharmacy “Iuliu Hațieganu”, Louis Pasteur street 6, 400349 Cluj-Napoca, Romania; catanaandreea@gmail.com (A.C.); ivpop.cj.ro@gmail.com (I.V.P.)

**Keywords:** adiponectin, tumor necrosis factor-alpha, type 1 diabetes mellitus

## Abstract

Type 1 diabetes mellitus (T1DM) is a complex condition caused by the destruction of pancreatic beta cells by autoimmune mechanisms. As a result, insulin deficiency and subsequent hyperglycemia occur. The aim of the present study is to investigate the role of adiponectin and tumor necrosis factor alpha (TNF-α) in the development of T1DM. The study is designed as an observational case-control study, involving 52 diabetic patients and 66 controls. Z scores for Body Mass Index (BMI), weight, height, and adiponectin and TNF-α serum levels were assessed in both groups. The T1DM group had significantly higher TNF-α levels and a significantly higher proportion of high-risk patients for inflammation based on TNF-α values as compared to the control group, while both groups had statistically similar adiponectin levels and a similar proportion of high/medium-risk patients based on adiponectin values. TNF-α plays a significant role in the pathogenesis and evolution of T1DM and it may represent an additional marker of disease progression, as well as a potential target of immunotherapeutic strategies. In the present study, no statistically significant differences were recorded in adiponectin levels neither in diabetic patients and controls, nor in high/medium severity risk diabetic patients.

## 1. Introduction

Type 1 diabetes mellitus (T1DM) is a multifaceted autoimmune metabolic disorder characterized by chronic idiopathic or immune-mediated, selective, specific destruction of pancreatic beta cells, leading to partial or, in most cases, total insulin deficiency and development of hyperglycemia [1,2]. It results from the interaction of three main factors: genetical, environmental, and immunological. Environmental trigger factors and insults in early life can activate self-targeting immune cascades. Cytokines and chemokines have a significant role in the stimulation, regulation, and intercellular signaling of immune cells, mediating insulitis, and beta cell destruction by islet autoantibodies [2,3,4,5,6].

Increasing prevalence of childhood obesity is strongly correlated with obesity in adulthood. Obese children are at a high risk of metabolic syndrome, cardiovascular diseases (CVD), and increased morbidity and mortality rates in their adult lives [7]. The adipose tissue is not only an inert repository of excess energy, with mechanical or temperature regulation functions, but also a complex, highly active metabolic endocrine organ [8]. It secretes numerous proinflammatory cytokines, named adipokines. Adiponectins are a peptide produced in white adipose tissue and in hepatocytes during stress. High adiponectin levels are associated with a markedly reduced relative risk of type 2 diabetes mellitus (T2DM). Measurement of adiponectin and leptin levels, as well as of adiponectin/leptin ratio may be useful tools in the differential diagnosis between T1DM and T2DM in pediatric patients [4,9,10,11,12].

Assessment of the role of inflammation mediated by acute phase proteins and cytokines in T1DM revealed that it can lead either to prevention or to promotion of diabetes. Tumor necrosis factor alpha (TNF-α) is a potent proinflammatory cytokine and immunomodulator produced by activated macrophages, monocytes, CD4+ lymphocytes, natural killer cells, neutrophils, mast cells, eosinophils, and neurons. It is involved in a variety of metabolic disorders such as T1DM, T2DM, and obesity. It blocks the action of insulin, causing insulin resistance. In humans, serum concentration of TNF-α is elevated in T2DM, being associated with impaired glucose tolerance, enhanced insulin resistance, islet dysfunction, and increased T2DM risk. T1DM patients also have significantly elevated serum levels of TNF-α as compared to healthy controls [4,13].

From a genetic point of view, T1DM is characterized by complex and unique interactions between enzyme systems: G alleles of TNF-α act as a protective factor in T1DM and T1DM patients show a higher TNF-α gene promoter methylation comparatively with healthy subjects [14].

Children and young adults are especially vulnerable to complications of several disorders such as diabetes, obesity, atherogenic dyslipidemia, and CVD (stroke and ischaemic heart disease). Chronic hyperglycemia in T1DM causes blood vessel damage, leading to chronic microvascular complications (nephropathy, retinopathy, and neuropathy) as well as to macrovascular complications (CVD). Microvascular complications can already be found in children and adolescents who have T1DM for at least five years. Moreover, these complications often progress more rapidly in children than in adults.

Diabetic ketoacidosis (DKA) is an acute, potentially life-threatening complication of diabetes, which remains the leading cause of hospitalization, morbidity, and mortality in children with T1DM. Cytokine release during DKA may be responsible for subclinical brain edema and interstitial lung edema.

Poor diabetes control is associated with higher adiponectin levels, while in obesity and dyslipidemia, lower concentrations of adiponectin and higher levels of inflammatory and endothelial biomarkers are recorded. Furthermore, increased concentrations of adiponectin in T1DM patients are independently associated with all-cause and cardiovascular mortality. Serum TNF-α level is higher in T1DM patients with microvascular complications [11,15,16,17,18]. These changes predispose to markedly increased cardiovascular morbidity and mortality in T1DM patients with poor disease control.

Although lifelong administration of exogenous insulin can help to balance to a certain degree glucose homeostasis in T1DM patients, currently there are no effective curative therapies available for this disease. Moreover, at the time of diagnosis, 80–90% of the pancreatic beta cell mass has been already lost. Furthermore, diabetes, its complications, and treatment seriously affect the quality of life of these patients and impose a large economic burden on the national healthcare system [13,19].

With the prevalence of T1DM increasing worldwide, there is a great need for more efficient means for early prediction, monitoring of progression, stratifying high-risk population for intervention, as well as for assessing the efficacy of novel therapies and eventually for preventing or reversing the disease and its complications as early as possible [5].

The aim of this study was to investigate the role of some specific cytokines such as adiponectin and TNF-α in the pathogenesis and evolution of T1DM in children and adolescents, by searching possible correlations between laboratory values and risk of T1DM, as well as to determine whether there is a link between serum levels of certain biomarkers and Z scores for weight, height, and BMI, age at the onset of the disease, association of other autoimmune disorders and onset of complications in T1DM pediatric patients.

## 2. Materials and Methods

### 2.1. Ethics Statement

This study was approved by the Ethical Committee of University of Medicine and Pharmacy “Iuliu Hatieganu” from Cluj-Napoca, Romania, (315, approved on 2 June 2015). Before enrollment in the study, each patient’s legal representatives signed an agreement to participate in this study. The present study was conducted in accordance with the Declaration of Helsinki.

### 2.2. Study Design

We performed a cross sectional, observational, case-control study comparing children and adolescents with T1DM to otherwise healthy controls. Patients were included from the electronic records of our institution: Pediatrics Clinic I of the Pediatric Emergency Hospital Cluj-Napoca, Romania.

The study group included 52 patients diagnosed with T1DM, (42.3% males, 57.7% females), aged between 1–18 years, on medical observation at Pediatrics Clinic I. Diagnosis of T1DM was based upon both classical clinical criteria: polyuria, nicturia, polydipsia, polyphagia, weight loss; and biochemical criteria: hyperglycemia > 126 mg/dL (baseline value,) or >200 mg/dL (random value, recorded after meals), glycated hemoglobin (HbA1C) > 6.5%, glicosuria or/and ketonuria [20,21].

The control group comprised of 66 subjects (51.5% males and 48.5% females), aged between 1–18 years, admitted to the same institution for different complaints, unrelated to diabetes, inflammation mediated and autoimmune diseases. Patients with other types of diabetes (T1DM, T2DM, genetic forms of diabetes—MODY), as well as patients with basal hyperglycemia, decreased glucose tolerance or symptoms resembling diabetes, were excluded from the control group.

Based on reference values of our laboratory, patients were classified in two categories: at low risk for atherosclerosis and insulin resistance if adiponectin level was >10 μg/mL and TNF-α level was ≤8.1 pg/mL or at high risk otherwise.

### 2.3. Patient Data Collection

Z scores for BMI, weight, and height were calculated by using either the ‘anthro’ R package (https://CRAN.R-project.org/package=anthro) with 2006 references for patients aged between 0–5 years or the R tools provided by WHO (https://www.who.int/growthref/tools/) with 2007 references for patients aged between 5–18 years.

Blood pressure was measured after at least 5 min of rest, with a standard mercury sphygmomanometer. Obtained values were interpreted according to charts for age and gender.

For measurement of plasma levels of adiponectin and TNF-α, 10 mL venous blood was collected after overnight fasting and previously to insulin administration in diabetic patients in vacutainers without anticlotting agent but with separating gel kept at a temperature of 4 °C until serum was separated by centrifugation. Serum samples were processed for adiponectin by Enzyme-Linked-Immuno-Sorbent-Assay (ELISA) method with Automatic Elisa Agility Dynex System equipment. For assessment of TNF-α level, the previously frozen serum sample was processed immunochemically using detection by chemiluminescence immunoassay (CLIA) with Immulite 1000 Immunoassay System (Siemens Healthcare GmbH, Erlangen, Germany).

For measurement of glycated hemoglobin level by High-Performance Liquid Chromatography (HPLC), 2 mL venous blood was collected in vacutainers with EDTA (Ethylenediaminetetraacetic acid). The sample was processed by D10 Hemoglobin Testing System (Bio-Rad Laboratories, Dubai, United Arab Emirates).

An additional 5 mL of blood was required for measurement of basal blood glucose, alpha 1 antitrypsin, cholesterol, high-density lipoprotein cholesterol (HDL cholesterol), and triglyceride levels by spectrophotometry using a Konelab Prime 60i Biochemical Analyzer (Thermo Fisher Scientific, Waltham, MA, USA).

For genetic extraction, 2 mL of peripheralvenous blood was collected in vacutainers with EDTA. The blood sample was kept at 4 °C until DNA extraction was performed using a genomic DNA extraction kit (Wizard DNA Extraction Kit, Promega Corporation, Madison, WI, USA). DNA concentration was tested spectrophotometrically and validation was followed by subsequent genotyping.

Adipo Q 276 G>T genotyping was based on PCR-RFLP protocol, and highlighted 3 possible genotypes: GG, TT, and GT. M1/T1 glutation S transferase genotyping followed a multiplex PCR protocol and null alleles of M and T alleles were identified as absence of amplicons in the reaction. TNFα genotyping was performed using a PCR-RFLP protocol, followed by electrophoretic analysis (Metaphore) with 3 different genotypes: A1A1, A1A2, and A2A2 homozygous mutant, respectively.

Hemolyzed or lipemic serum samples were not processed.

### 2.4. Data Analysis

Each patient’s data were manually collected into a spreadsheet for prepossessing then imported into R 3.6.2 for statistical analysis [22]. We used descriptive methods as appropriate: numeric and percent frequencies, means ± standard deviation and medians with extreme values. We also reported geometric means and geometric standard deviations for adiponectin and TNF-α values. We compared the T1DM group to the control group using univariate methods: odds ratio (OR) with Fisher test for binary variables, Cramér’s V coefficient with Chi² test for other categorical variables, T and Mann–Whitney test (MW) for normally and non-normally distributed numerical variables, respectively. Correlations between adiponectin or TNF-α and other numerical variables were studied using Spearman’s rho coefficients for all patients as well as separately for both groups. We used logistic models for OR (95% CIs) of T1DM by adiponectin or TNF-α, unadjusted (models 1, used as reference for deviance tests with the multivariate models) and adjusted for BMI Z score (models 2), age at inclusion and sex (models 3), and all three covariates combined (models 4). Both adiponectin and TNF-α values were transformed to base 2 logarithms prior to logistic regression, therefore odds ratios are referenced to every doubling of the original values.

## 3. Results

The study group included 52 T1DM patients (mean age at inclusion 11.94 ± 4.45 years) and 66 controls (mean age at inclusion 11.09 ± 4.82 years). T1DM patients had significantly higher mean Z scores for weight compared to control patients (0.898 ± 1.24 vs. −0.317 ± 1.05, *p* < 0.001) as well as for BMI (0.298 ± 1.15 vs. −0.459 ± 1.61, *p* = 0.011). There were no statistically significant differences of systolic and diastolic values of blood pressure between T1DM patients and controls (Table 1).

Both groups had statistically similar adiponectin levels (μg/mL, median: 13.57, range = 6.82 to 26.61 vs. 13.85, range = 7.05 to 22.31, *p* = 0.774) and a similar proportion of high or medium risk patients based on adiponectin values (19.2% vs. 15.2%, OR = 1.33, *p* = 0.625). Only 9 T1DM patients (17.3%) had adiponectin levels < 10 μg/mL, corresponding to medium severity risk for atherosclerosis and just one of these patients had adiponectin levels < 7 μg/mL, corresponding to high risk for atherosclerosis.

T1DM patients had significantly higher TNF-α levels as compared to controls (pg/mL, median: 9.7, range = 5.3 to 27.1 vs. 7.1, range = 5.6 to 15.5, *p* < 0.001) and a significantly higher proportion of high-risk patients based on TNF-alpha values (80.8%, vs. 12.1%, OR = 30.45, *p* < 0.001).

Fourty-two patients with diabetes (80.67%) displayed high concentrations (≥8.1 pg/mL) of TNF- α as compared to controls (Table 2).

**Figure 1:**
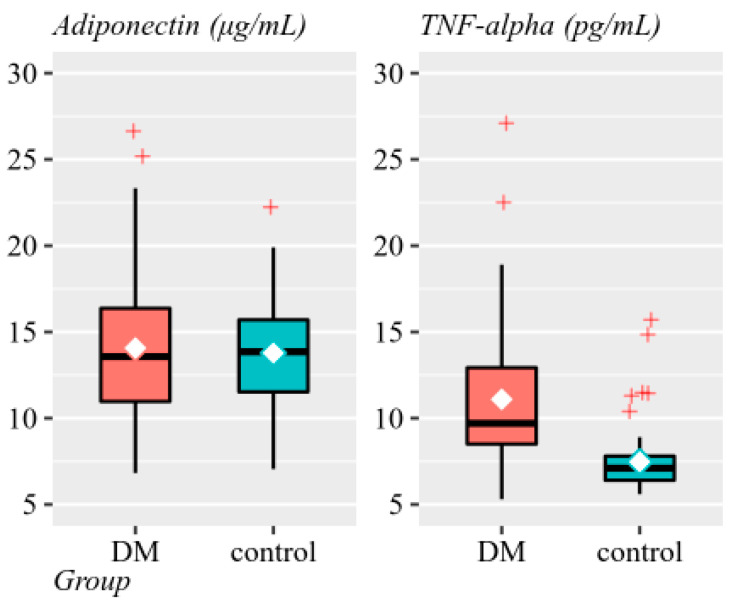
Adiponectin (μg/mL) and TNF-α (pg/mL) values by groups (◆ mean, — median, + outliers).

Since both adiponectin and TNF-α distributions are skewed, we also provided geometric means and standard deviations (Figure 1).

T1DM patients also had significantly higher total cholesterol levels comparatively with controls (mg/dL, median: 169, range = 111:353 vs. 155, range = 99:200, *p* < 0.001) and glycosylated hemoglobin (HbA1C) (%, median: 8.7, range = 6.7:15.4 vs. 4.85, range = 4:5.9, *p* < 0.001), as depicted in Table 3.

With limited data available, none of the studied genes showed any significant differences in genotype distributions between cases and controls (Table 4).

T1DM patients have received 0.96 ± 0.26 IU insulin/kg/day, administered either in 4 injections/day (48.1%), 5 injections/day (44.2%), or by insulin pump (7.7%).

Regarding insulin therapy schedules in T1DM patients, no statistically significant differences have been recorded between 4 daily doses, 5 daily doses regimens, and administration by insulin pump (Table 5).

In 32.7% of T1DM patients, other autoimmune diseases had been also diagnosed: either celiac disease (CD), autoimmune thyroiditis (AIT), or both (*p* < 0.001, with a median age of 9 years at onset).

On the contrary, no autoimmune diseases were recorded in the control group, excepting a patient with positive ANA antibodies (found at the age of 7 years), but without clinical manifestations of disease.

Neuropathy, nephropathy, and retinopathy were only found in T1DM patients (in 36.5, 11.5, and 4%, respectively), but not in controls (Table 6).

In T1DM patients, other frequent complications were the following, in decreasing range of frequency: insulin lipodystrophies (67.3%), dawn phenomenon (42.3%), and dyslipidemias (26.9%). Most patients had only one complication (40.37%), while 2, 3, and 5 complications were recorded in 25, 19.23, and 1.92% of the cases, respectively. In 13.46% of the study group members, no complications have yet occurred (Table 7).

**Figure 2:**
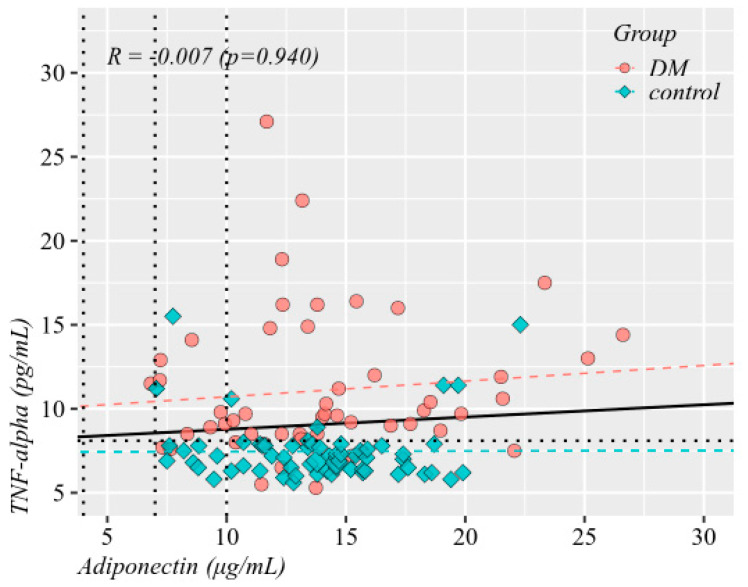
Correlation matrix of adiponectin and TNF-α values with several other parameters (Spearman’s R coefficients, statistically insignificant values crossed out).

**Figure 3:**
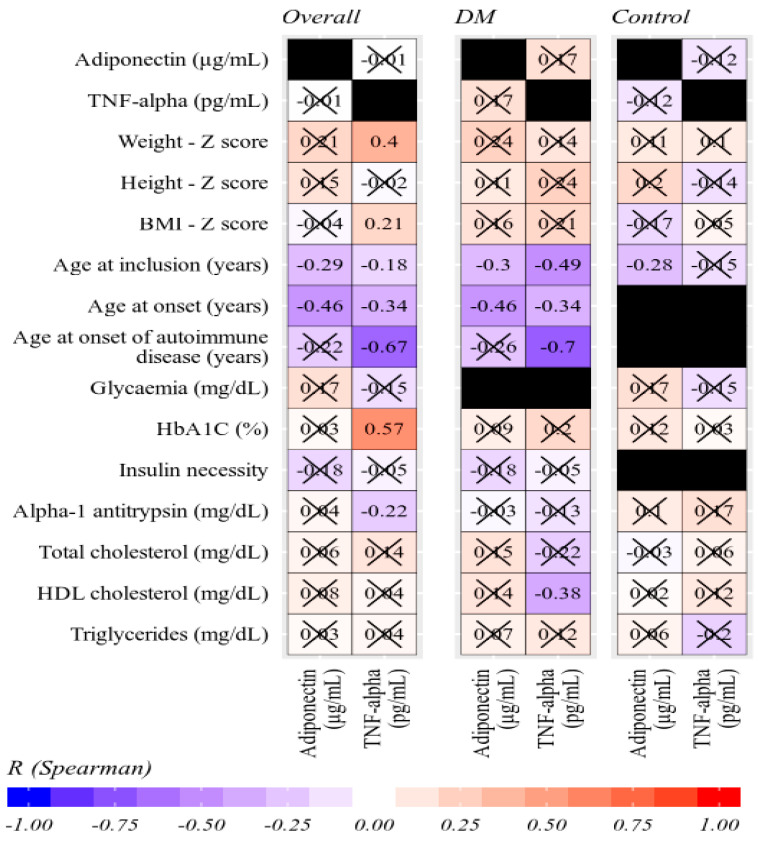
Correlation between adiponectin and TNF-α (Spearman’s R coefficient). Reference values marked by dotted lines at 8.1 pg/mL for TNF-α and at 4, 7, and 10 μg/mL for adiponectin (Spearman’s R coefficients, statistically insignificant values crossed out, black boxes—parameters could not be corelated between the two groups).

Adiponectin values were not significantly correlated to TNF-α values, in neither of the 2 groups. In T1DM patients, higher adiponectin and TNF-α values were significantly correlated to younger ages at inclusion and at onset of T1DM. In addition, TNF-α showed significant negative correlations with age at onset of autoimmune disease and with HDL cholesterol levels. These correlations decreased to statistical insignificance in controls, with the exception of adiponectin at age of inclusion, which decreased but remained statistically significant. Overall, HbA1C was significantly correlated with TNF-α, but not in separate groups because HbA1C values formed clusters with weaker intra-cluster correlations as compared to between-clusters (Figure 2 and Figure 3).

### Multivariate Models

Odds of T1DM significantly increased with every doubling of TNF-α values, unadjusted (OR = 42.40, 11.04–221.00) as well as adjusted for BMI Z scores (OR = 36.36, 9.52–187.95); age and gender (OR = 129.30, 24.7 to 999.2); and BMI, age, and gender (OR = 118.16, 22.13 to 934.82).

**Figure 4:**
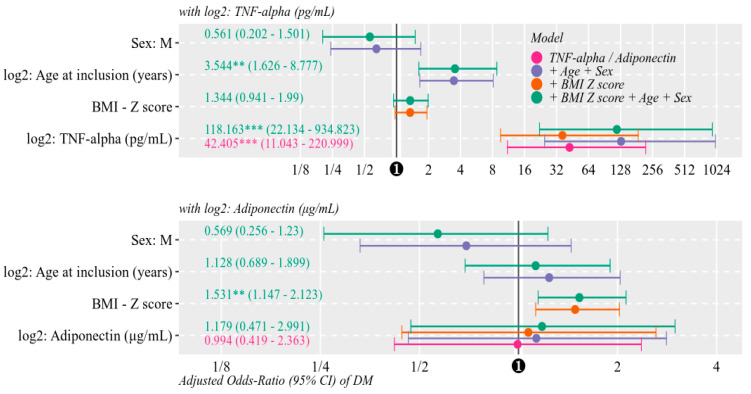
Logistic models for odds-ratios of T1DM by adiponectin or TNF-α, unadjusted (purple) and adjusted for BMI Z score (orange), age at inclusion and gender (blue), and all three covariates combined (green). Adiponectin, TNF-α and age were transformed to base 2 logarithms, therefore odds ratios are referenced to every doubling of original values. (*p*-value = ⁺: <0.10, **: < 0.01, ***: <0.001).

Odds of T1DM did not increase with every doubling of adiponectin values, unadjusted (OR = 0.99, 0.42–2.36); or adjusted for BMI Z scores (OR = 1.07, 0.44 to 2.62); age and gender (OR = 1.13, 0.46 to 2.82); or BMI, age and gender (OR = 1.18, 0.471to 2.991) (Figure 4).

## 4. Discussion

As far as we know, the present study is the first in Romania with the main goal to assess the role of adiponectin and TNF-α in the pathogenetic pathways and outcome of T1DM in pediatric patients.

Our results, regarding the role of adiponectin in the pathogenesis and evolution of T1DM, reached similar conclusions to other authors, but in discordance with other literature data, possibly due to the limits of the present study.

Similar findings as in the present paper were recorded in another study where no difference had been found in adiponectin levels between diabetic patients and healthy controls. Moreover, serum adiponectin levels were not correlated with diabetes duration or HbA1C, but they were negatively correlated with BMI [23,24].

Contradictive results were also reported by other investigators. Several studies revealed alteration in adipokine phenotype, which proved to be more proinflammatory at onset. This change in the type of action may be a consequence of several factors associated with diabetes such as insulin sensitivity, immune activity, obesity, physical activity, or diet. A recent study concluded that predicted adiponectin concentrations at 1 month, 6 months and 12 months of T1DM follow up, respectively, were associated with distinct progression patterns of T1DM after establishment of diagnosis. Furthermore, lower adiponectin concentrations were found in recent years in T1DM patients as compared to their healthy siblings, as well as in the first 2 days after initiation of insulin treatment as compared to later course of therapy. There was a negative correlation of adiponectin level with age and a significant positive correlation with BMI [8,25,26].

On the other hand, serum adiponectin was higher in T1DM and lower in T2DM, as compared to the group of subjects with normal glucose tolerance, so an adiponectin cut point of 5.1 µg/mL may be a useful tool for differentiating T1DM and T2DM, which is necessary for optimum treatment [27].

Significantly increased serum adiponectin in T1DM pediatric patients compared with controls were found in two different research papers [28,29]. Adiponectin serum concentrations were not related to body fat content. No correlations have been identified between adiponectin level and any of the analyzed parameters of the disease course: duration, metabolic control (HbA1C), triglycerides, serum levels of total cholesterol and free fatty acids, daily insulin dose [29].

Both at onset and during the course of T1DM, adiponectin concentrations were significantly increased comparatively with controls, but this change was not accompanied by plasma lipid alterations, apart from increased free fatty acid levels in diabetic patients [30]. Serum levels of adiponectin were higher in poorly controlled than in well-controlled disease in T1DM patients and in controls. In consequence, it can be used as a reliable marker of metabolic control state in diabetic patients [31].

In the Wisconsin Diabetes Registry Study, the following conclusions were drawn: adiponectin level decreased with age, being more stable after the age of 20 years, females had higher adiponectin levels than males, and adiponectin was strongly correlated with waist circumference, weight, BMI, HbA1C, intensive insulin management, insulin pump use, and log insulin dose [32]. Adiponectin levels increased significantly following insulin therapy in the first five days and then had stabilized during the remission period in the third month after disease onset. Also, subjects with recent onset of T1DM, with the fewest autoantibodies, had the lowest adiponectin level, supporting the concept that insulin-resistant subjects may present with clinical T1DM in earlier stages of beta cell damage [33].

Another research paper found that adiponectin levels were lower, while serum levels of total cholesterol, LDL-cholesterol, triglycerides, and insulin were higher in obese children versus healthy subjects. This suggests that out of all adipocytokines, adiponectin is the best indicator of metabolic syndrome and monitoring adiponectin level may allow early intervention in obese children with metabolic syndrome [34].

Similarly to the results of another study, children and adolescents on medical treatment for obesity, after a one-year follow up period, did not improve their adiponectin profile, even though the mean BMI Z scores lowered and HDL-cholesterol increased over this period [12].

Another marker assessed in our analysis was TNF-α.

Results obtained are in accordance with several animal models and genetic studies, which highlight the important role of autoimmune-based mechanisms in T1DM. Alterations of regulatory T cells and imbalance between CD^4^+ T-helper 1 and 2 cells may contribute to ineffective suppression of proinflammatory cytokines in T1DM. Infiltrative autoreactive T cells cause pancreas islet inflammation and subsequent beta cell destruction. Cytokine profiles in T1DM patients are characterized by significantly increased TNF-α, which has a strong correlation with a patient’s age and blood glucose level [28,35,36,37,38]. These conclusions are sustained by other investigators who reported higher TNF-α levels in T1DM pediatric patients, a tendency towards higher TNF-α gene expression, and higher methylation levels in the TNF-α gene promoter region [14].

In another research study, significantly increased serum levels of TNF-α were found in T1DM patients as compared to controls. A positive correlation with BMI and obesity was also recorded in these patients, as an indicator of hyperinsulinemia, which confirmed that proinflammatory cytokines may be increased by hyperglycemia in subjects with impaired glucose tolerance [19].

In newly diagnosed T1DM patients, TNF-α concentrations were higher in males than in females, in accordance with our results, TNF- α levels being also increased in the islet autoantibody-positive T1DM group. Similarly to our findings, TNF-α level decreased with age and duration of disease [39]. Serum levels of TNF-α were higher in obese children than in healthy subjects and they were not correlated with central and global obesity [12,34].

Regarding the evolution and potential chronic complications of T1DM, these occur by angiopathic mechanisms and appear 10–20 years following the onset of the disease. Late-stage microangiopathic and macroangiopathic complications can start early in childhood, mainly in patients with poorly controlled diabetes. These complications can be either prevented or delayed by good metabolic control. It is worth remembering that the onset of these complications can already occur in childhood, and their close monitoring and early treatment and even better, their prevention, if possible are of tremendous importance.

In the present study, adiponectin levels were not significantly correlated with TNF-α levels in either of the two study groups. In T1DM patients, higher adiponectin and TNF-α values were significantly correlated with younger age at onset of diabetes and they also showed significant negative correlations with age at onset of the autoimmune disease and HDL-cholesterol. Overall, HbA1C was significantly correlated with TNF-α, but not in separate groups. These findings are similar to several previous reports [32,40,41].

Serum adiponectin and TNF-α levels accurately predicted subclinical hyperlipidemia and/or diabetes in an animal model. Subsequently, these molecules may serve as prognostic predictors in obesity and/or T1DM [42].

Obesity increases the risk of CVD and other complications in T1DM. A recent research paper concluded that obese children with recent autoimmune T1DM had significantly higher serum TNF-α and lower total adiponectin levels, which may contribute to the development of CVD and other diabetic complications [43]. Moreover, soluble TNF-α receptors negatively correlated with insulin sensitivity, while plasma levels of adiponectin negatively correlated with insulin, HOMA-IR, and triglycerides, and positively correlated with insulin sensitivity [7].

Significant biomarkers of inflammation and endothelial dysfunction are present in young patients with T1DM, obesity, and dyslipidemia. Adiponectin correlated negatively with inflammatory biomarkers in patients with diabetes, while HbA1C > 8.0% (estimated average blood glucose >10 mmol/L) was correlated with higher adiponectin levels. On the other hand, obesity was associated with lower adiponectin and higher TNF-α concentrations, while HDL < 1.02 mmol/L was associated with higher TNF-α and lower adiponectin levels [11].

Pediatric patients with T1DM, regarding their higher future risk of CVD, are characterized by higher concentrations of protective adiponectin and paradoxically lower blood concentrations of some other possible markers of atherosclerosis, comparatively with healthy children [44]. These findings are in contradiction with the results of other studies, which indicate that excess adiposity is associated with increased cardiovascular risk in youth with T1DM, but it is not significantly correlated with adiponectin [24,40].

Another study demonstrated that diabetic pediatric patients without clinical manifestations of CVD had elevated adiponectin concentrations, increased arterial stiffness, reduced arterial distensibility, and arterial compliance as signs of alteration of arterial wall function [45].

Another research study revealed that in children and adolescents with relatively well-controlled glycemia, there were no abnormalities of adiponectin as a marker of CVD, but on the other hand, adiponectin level was negatively correlated with skinfold thickness and positively correlated with daily energy intake and saturated fat intake, suggesting that increased serum adiponectin could protect patients against the risk of CVD [46].

Other authors found no relevant correlation between adiponectin and progression of pulse wave velocity (PWV). In a study involving diabetic adolescents, significant progression of arterial stiffness, measured by PWV, had been revealed [47].

A previous study’s contribution of adiponectin to arterial stiffness appeared to be masked by other cardiovascular risk factors such as age, male gender, high blood pressure, obesity, and total cholesterol in adolescents with T1DM. Serum adiponectin concentrations were not independently associated with non-ultrasound measurements of arterial stiffness [41,47].

A group of authors reported high serum adiponectin levels in children and adolescents with T1DM and low levels of the same biomarker in patients with diabetic retinopathy [40].

A study that included children and adolescents with T1DM (divided into complicated and not complicated forms of disease) compared to healthy subjects, demonstrated that elevated adiponectin level in T1DM patients was correlated directly with HbA1C and creatinine; thus, it indicated poor glycemic control and development of complications, especially nephropathy [48].

Another group of authors demonstrated that serum TNF-α concentrations over 1.7 pg/mL may point out the presence of diabetic microangiopathy in pediatric patients with T1DM [18].

In other studies, TNF-α has also been shown to be increased in patients with microvascular complications (microalbuminuria 30%, neuropathy 17.1%, retinopathy 12.9%). At the same time, alteration in the number of regulatory T cells expressing TNF-α receptor type 2 in T1DM was associated with augmented inflammation and poor glycemic control [35].

In a research study involving a group of diabetic adolescents, who were at least two years post-diagnosis of T1DM, TNF-α as a biomarker of inflammation was not significantly different between subjects with HbA1C ≤ 8.5% and those with HbA1C ≥ 9.6% [49].

Hypertension is a classical risk factor for peripheral vessel damage and endothelial dysfunction. TNF-α concentration is significantly higher in the group of patients with T1DM and high blood pressure. These two combined risk factors can cause vascular involvement early in the course of the disease [17].

Another study reported that adults with T1DM had higher adiponectin levels than their non-diabetic peers, and elevated adiponectin at baseline was independently associated with greater odds for developing early diabetic renal disease over a period of six years, while lower adiponectin concentrations were associated with CVD among patients with T1DM [50].

Limits of the present study consist in the small number of included subjects, and the relatively restricted geographic area they were originating from. In the same time, because the patients included in the study group were young, only a few of them had already developed changes in adiponectin levels, which did not always allow statistically significant conclusions. These restrictions can explain discordances with results of other similar research.

## 5. Conclusions

Several disease-promoting cytokines known as markers of inflammation or adiposity are dysregulated in T1DM children and adolescents. Our findings showed that diabetic patients and healthy controls had statistically similar adiponectin levels and there was a similar proportion of high/medium-risk patients based upon adiponectin values. TNF-α levels were significantly higher in T1DM subjects comparatively with the control group and a significantly higher proportion of high-risk patients based upon TNF-α values were recorded in the T1DM group. Moreover, in T1DM patients, higher adiponectin and TNF-α values were significantly correlated with younger ages at inclusion and at the onset of diabetes, and they also showed significant negative correlations with age at the onset of autoimmune diseases and HDL-cholesterol levels. Therefore, TNF-α may serve as an additional marker of diabetes and it can provide valuable information about the pathogenetic pathways and regulation of the disease process, leading to development of new immunotherapeutic strategies.

## Figures and Tables

**Table 1:** Comparative demographic and anthropometric description of the two groups.

Parameters	Details	Cases	Controls	Total	Statistics
**Group**		52 (44.1%)	66 (55.9%)	118	
Age at inclusion (years)	μ ± SD	11.94 ± 4.45	11.09 ± 4.82	11.47 ± 4.66	*T*-test: *p* = 0.327
Age at onset (years)	μ ± SD	6.67 ± 3.82	N/A	6.67 ± 3.82	MW: *p* = 0.019
Gender n (%)	Female	30 (57.7%)	32 (48.5%)	62 (52.5%)	OR = 1.45 [0.70, 3.01] (*p* = 0.357)
	Male	22 (42.3%)	34 (51.5%)	56 (47.5%)	
Patient’s residence area	rural	24 (46.2%)	26 (39.4%)	50 (42.4%)	OR = 1.32 [0.63, 2.75] (*p* = 0.574)
	urban	28 (53.8%)	40 (60.6%)	68 (57.6%)	
Weight–Z score	μ ± SD	0.898 ± 1.24	−0.317 ± 1.05	0.158 ± 1.27	*T*-test: *p* < 0.001
Height–Z score	μ ± SD	0.426 ± 1.27	0.409 ± 1.26	0.417 ± 1.26	*T*-test: *p* = 0.945
Body mass index–Z score	μ ± SD	0.298 ± 1.15	−0.459 ± 1.61	−0.125 ± 1.47	Welch: *p* = 0.004
Systolic blood pressure (mmHg)	μ ± SD	102.71 ± 12.2	102.04 ± 12.6	102.26 ± 12.4	MW: *p* = 0.377
Diastolic blood pressure (mmHg)	μ ± SD	57.08 ± 10.1	57.71 ± 9.64	57.28 ± 9.86	MW: *p* = 0.603

μ ± SD = mean (standard deviation); Welch = Welch *T*-test (not assuming equal variances); OR/RR = odds-ratio/risk-ratio [95% CI = 95% confidence interval] and *p* value from Fisher test); MW = Mann–Whitney test; SE = standard error.

**Table 2:** Adiponectin (μg/mL) and TNF-α (pg/mL) values by group, as well as by risk of insulin resistance.

Parameters	Details	Cases	Controls	Statistics
**Group**		52 (44.1%)	66 (55.9%)	
Adiponectin (μg/mL)	μ ± SD	14.07 ± 4.69	13.78 ± 3.39	MW: *p* = 0.774
	M (range)	13.57 (6.82:26.61)	13.85 (7.05:22.31)	
	Gμ ± SD	13.33 ± 1.4	13.34 ± 1.3	
Insulin resistance risk	medium-high	10 (19.2%)	10 (15.2%)	OR = 1.33 [0.51, 3.49] (*p* = 0.625)
	low	42 (80.8%)	56 (84.8%)	
TNF-alpha (pg/mL)	μ ± SD	11.09 ± 4.21	7.47 ± 1.85	MW: *p* < 0.001
	M (range)	9.7 (5.3:27.1)	7.1 (5.6:15.5)	
	Gμ ± SD	10.46 ± 1.4	7.30 ± 1.22	
Inflammation risk	high	42 (80.8%)	8 (12.1%)	OR = 30.45 [11.08, 83.68] (*p* < 0.001)
	normal	10 (19.2%)	58 (87.9%)	

μ ± SD = mean (standard deviation); Gμ ± SD = geometric mean (geometric standard deviation); M (range) = median (min:max); MW = Mann–Whitney test; OR/RR = odds-ratio/risk-ratio [95% CI] and *p* value from Fisher test).

**Table 3:** Other laboratory values by groups.

Parameters	Details	Cases	Controls	Statistics
**Group**		52 (44.1%)	66 (55.9%)	
Triglycerides (mg/dL)	μ ± SD	89.23 ± 51.7	73.30 ± 32.5	MW: *p* = 0.109
	M (range)	72 (26:318)	66.5 (24:187)	
Total cholesterol (mg/dL)	μ ± SD	177.71 ± 42.7	155.59 ± 23.1	MW: *p* = 0.001
	M (range)	169 (111:353)	155 (99:200)	
HDL cholesterol (mg/dL)	μ ± SD	55.44 ± 10.3	52.36 ± 10.1	MW: *p* = 0.126
	M (range)	54 (31:84)	51.5 (31:78)	
HbA1C (%)	μ ± SD	9.03 ± 1.79	4.83 ± 0.477	MW: *p* < 0.001
	M (range)	8.7 (6.7:15.4)	4.85 (4:5.9)	

μ ± SD = mean (standard deviation); M (range) = median (min:max); MW = Mann-Whitney test.

**Table 4:** Univariate statistics by groups of genotypes for several relevant genes, where available.

Variable	Details	DM	Control	Statistics
**Group**		52 (44.1%)	66 (55.9%)	
276G/TAdiponectin	GG	8 (15.4%)	3 (23.1%)	V = 0.17 (*p* = 0.390)
	GT	31 (59.6%)	5 (38.5%)	
	TT	13 (25.0%)	5 (38.5%)	
Adipo Q genotype	mutant	16 (47.1%)	31 (49.2%)	V = 0.07 (*p* = 0.774)
	heterozygote	15 (44.1%)	24 (38.1%)	
	wild-type	3 (8.8%)	8 (12.7%)	
GSTM genotype	M-	22 (42.3%)	6 (46.2%)	OR = 0.86 [0.25, 2.90], phi = 0.03 (*p* > 0.999)
	M+	30 (57.7%)	7 (53.8%)	
GSTT genotype	T-	17 (32.7%)	4 (30.8%)	OR = 1.09 [0.29, 4.06], phi = 0.02 (*p* > 0.999)
	T+	35 (67.3%)	9 (69.2%)	
TNF-alfa genotype	A1A1	39 (75.0%)	9 (69.2%)	V = 0.09 (*p* = 0.761)
	A1A2	12 (23.1%)	4 (30.8%)	
	A2A2	1 (1.9%)	0	

OR/RR = odds-ratio/risk-ratio [95% CI] and *p* value from Fisher test); V = Cramér V (*p* value from Chi^2^ test); GSTM = glutathione S transferase isoforms mu; GSTT = glutathione S transferase isoforms theta.

**Table 5:** Insulin therapy in Type 1 diabetes mellitus (T1DM) patients.

Parameters	Details	Cases	Controls	Statistics
**Group**		52 (44.1%)	66 (55.9%)	
Insulin dose (IU/kg/day)	μ ± SD	0.961 ± 0.26	N/A	MW: *p* = 0.583
	M (range)	1 (0.36:1.51)	N/A	
Injections/day	4	25 (48.1%)	N/A	V = 0.23 (*p* = 0.265)
	5 insulin pump	23 (44.2%) 4 (7.7%)	N/A N/A	

μ ± SD = mean (standard deviation); M (range) = median (min:max); MW = Mann–Whitney test; V = Cramér V (*p* value from Chi^2^ test); IU = international units.

**Table 6:** Autoimmune diseases in diabetic patients.

Parameters	Details	Cases	Statistics
**Group**		52 (44.1%)	
Autoimmune disease	Yes	17 (32.7%)	OR = 64.58 [3.77, 106.01] (*p* < 0.001)
	AIT+CD	5 (9.6%)	
	AIT	10 (19.2%)	
	CD	2 (3.8%)	
	No	35 (67.3%)	
Age at onset of autoimmune disease (years)	μ ± SD	9.18 ± 4.03	MW: *p* = 0.493
	M (range)	9 (2:16)	

μ ± SD = mean (standard deviation); M (range) = median (min:max); MW = Mann–Whitney test; OR/RR = odds-ratio/risk-ratio [95% CI] and *p* value from Fisher test); CD: celiac disease; AIT: autoimmune thyroiditis.

**Table 7:** Complications in diabetic patients.

Parameters	Details	Cases	Statistics
**Group**		52 (44.1%)	
Retinopathy	minimal retinal changes	1 (1.9%)	V = NA (*p* > 0.999)
	mild diabetic nonproliferative retinopathy diabetica neproliferativa usoara	1 (1.9%)	
	No	50 (96.2%)	
Nephropathy	transient microalbuminuria	4 (7.7%)	V = NA (*p* > 0.999)
	incipient diabetic nephropathy	2 (3.8%)	
	No	46 (88.5%)	
Neuropathy	subclinical sensitive neuropathy	14 (27%)	V = NA (*p* > 0.999)
	sensitive neuropathy	4 (7.7%)	
	aggravated sensitive neuropathy	1 (1.9%)	
	No	33 (63.5%)	

V = Cramér V (*p* value from Chi^2^ test).
